# Can a theory-based educational intervention change nurses’ knowledge and attitudes concerning cancer pain management? A quasi-experimental design

**DOI:** 10.1186/1472-6963-13-328

**Published:** 2013-08-19

**Authors:** Markus Gustafsson, Gunilla Borglin

**Affiliations:** 1 School of Health Science, Blekinge Institute of Technology, Blekinge, SE-379 71, Sweden

**Keywords:** Attitude, Cancer pain, Evidence-based practice, Education, Experimental study, Implementation, Intervention, Knowledge, Nurses, Quasi-experimental design

## Abstract

**Background:**

Registered Nurses (RNs) play an important role in caring for patients suffering from cancer pain. A lack of knowledge regarding pain management and the RNs’ own perception of cancer pain could act as barriers to effective pain management. Educational interventions that target RNs’ knowledge and attitudes have proved promising. However, an intervention consisting of evidence-based practice is a multifaceted process and demands behavioural and cognitive changes to sustain the effects of the intervention. Therefore, our study aimed to investigate if a theory-based educational intervention could change RNs’ knowledge and attitudes to cancer pain and pain management, both four and 12 weeks after the start of the intervention.

**Methods:**

A quasi-experimental design with non-equivalent control groups was used. The primary outcome was measured using a modified version of the instrument Nurses’ Knowledge and Attitudes Survey Regarding Pain (NKAS) at baseline, four weeks and 12 weeks after the start of the intervention to evaluate its persistence. The intervention’s educational curriculum was based on the principles of Ajzen’s Theory of Planned Behaviour and consisted of interactive learning activities conducted in workshops founded on evidence-based knowledge. The RN’s own experiences from cancer pain management were used in the learning process.

**Results:**

The theory-based educational intervention aimed at changing RNs knowledge and attitudes regarding cancer pain management measured by primary outcome NKAS resulted in a statistical significant (p<0.05) improvement of total mean score from baseline to four weeks at the intervention ward.

**Conclusions:**

The findings of this study, suggest that a theory-based educational intervention focused at RNs can be effective in changing RN’s knowledge and attitudes regarding cancer pain management. However, the high number of dropouts between baseline and four weeks needs to be taken into account when evaluating our findings. Finally, this kind of theory-based educational intervention with interactive learning activities has been sparsely researched and needs to be evaluated further in larger projects.

**Trial registration:**

Clinical Trials. Gov: NCT01313234

## Background

Pain is experienced as one of the most feared and troublesome symptoms among patients with a cancer diagnosis [1] and approximately 50% of patients are expected to experience moderate to severe pain [1-3]. Cancer pain can result in difficulty performing normal daily activities [2], disruption in the patient’s sleep pattern and negative emotional experiences [4]. During the last century, there has been a substantial increase in knowledge of how to manage cancer pain effectively [5-8] and it is estimated that around 90% of cancer patients can achieve acceptable pain relief if they are offered adequate pain management [6]. Despite this advance in knowledge, cancer pain is still undertreated worldwide [1,2,9], highlighting the importance of implementation of evidence-based knowledge in cancer pain management.

Registered Nurses (RNs) play a crucial role in caring for patients’ suffering related to cancer pain. Nursing activities, such as regular evaluations of pain treatment, standardised pain assessments and the use of pharmacological and non-pharmacological pain interventions, are the core of effective pain management, especially when it concerns patients suffering from cancer pain [10-12]. In a systematic review investigating barriers to adequate cancer pain management, insufficient assessment of pain, patients’ reluctance to report pain and poor knowledge of pain management were found to act as barriers for both patients and professionals [13]. In addition, RNs’ knowledge of cancer pain and their attitudes to pain have been shown to be vital components in achieving optimal pain management [14-17]. An unwillingness to administer opioids and a lack of knowledge among RNs [18,19] would probably result in undertreatment and unnecessary suffering among patients. The literature review illustrates shortcomings in pain management among RNs, particularly when it concerns RNs’ evidence-based practice in cancer pain management and it would thus appear appropriate to explore how this important nursing activity can be improved.

One common strategy to improve patient care and its outcome, i.e. to work in accordance with evidence-based practice, is education-based intervention [20]. This kind of intervention could improve RNs’ knowledge and attitudes [15,21]. We now know that certain forms of educational interventions are effective in changing RNs’ knowledge and attitudes although it has been shown that the positive effects do not stand up to the test of time and even revert to pre-intervention levels after three months [14]. Knowledge translation can be a real challenge since it involves an actual cognitive change among the RNs to sustain what has been learned [22]. However, interactive educational activities that target small groups, i.e. workshops, have shown promise and an interactive learning design based on personal experiences can promote a positive change in behaviour [23,24]. This kind of approach can link learning activities to the actual problems experienced by the RNs, which can in turn lead to better sustainability of the RNs’ own knowledge [20].

Another area of importance is to take prospectively identified barriers into account when planning the intervention as this increases the likelihood of improving clinical practice [25]. One contributing barrier to bringing about change is how the RNs value the aims of the intervention, i.e. their attitudes. It is therefore important to assess and discuss both positive and negative attitudes to the components that make up the intervention in an attempt to increase the ambition among RNs to learn and perform the desired outcomes [26]. Consequently, it is vital to take into account how as humans we respond to demands for a change in behaviour in order to design interventions that increase knowledge and influence attitudes positively, the ultimate aim being to sustain the effects of the intervention.

The purpose of this study was to investigate if a theory-based educational intervention could change RNs’ knowledge and attitudes to cancer pain and pain management, both four and 12 weeks after the start of the intervention.

## Methods

### Design

In this study, a quasi-experimental design with non-equivalent control groups was used [27]. Pre-test measurement points for the primary outcome, the NKAS, were collected from the RNs at baseline (T1) and at post-test measurement points at four weeks (T2) and 12 weeks (T3) after the workshop. T1 was initiated before the theory-based educational intervention in order to evaluate the persistence of the intervention.

This study stems from the philosophy of pragmatism, where research is seen as something that should benefit the patients and that research should also be evaluated in compliance with practice [28]. The rationale for using a quasi-experimental design was to investigate the relationship between cause and effect of outcome variables and to account for the complexity of the nursing environment. For ethical, financial or legal reasons a true experimental design is thus not always the most suitable in this context [27]. Randomisation checks for selection bias, which is one of four biases [29], and removal of selection bias allow for a more pragmatic research design that is considered to be better suited in the context.

### Participants and study setting

The RNs were recruited in late autumn 2011 from two surgical wards at a hospital in southern Sweden that frequently care for patients with cancer. The research team carried out the assignment on either the intervention ward or the control ward. Each ward had 26 beds and each year they admitted around 820 cancer patients. The eligible RNs (n = 60) were all permanently employed RNs on both wards, about 30 on each, and they were expected to participate as the study was seen as a source of support in the RNs’ annual education programme and as a form of quality assessment. On the control ward patient’s received care as usual and the RNs only participated in the study by completing NKAS.

### Outcome assessment

Demographic information about the participating RNs was collected, including items related to gender, age, work experience as an RN, level of education and earlier pain education. The RNs’ knowledge and attitudes, the primary outcome, were measured using a modified version of the Nurses’ Knowledge and Attitudes Survey Regarding Pain (NKAS), consisting of 38 items and where each correct answer carries one point (total score 38 points). Although the original NKAS [30] consists of 40 items, two items were removed – 9 and 18 – as they were considered to represent the original instrument’s country of origin and were not applicable to the Swedish context. Items 1–22 are false-true statements and items 23–36 are multiple-choice questions with only one correct answer. Items 37–40 consist of two patient scenarios. Items 37–40 have a Likert-type scale [31], ranging from 0–10, but with only one number seen as a correct answer. Internal consistency [32] in earlier studies has ranged from 0.70 to 0.73 [33,34] and the test-retest reliability was r >0.80 [30]. The Swedish version of the NKAS in the present study displayed an internal consistency [32] of 0.70. Since the NKAS had not previously been tested in Swedish, an authorised translator conducted the back translation [35] from Swedish to English. The back translation was in accordance with the text in the original instrument. The primary outcome, the NKAS [30], was assessed on both wards simultaneously at T1, T2 and T3 in order to account for possible exposure bias [27].

### The theory-based educational intervention

**Figure 1:**
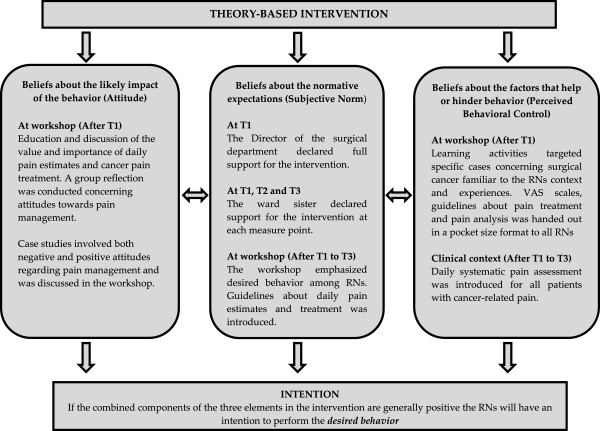
Overview of the theory-based intervention.

The educational intervention in this study (Figure 1) was based on the principles of Ajzen’s Theory of Planned Behaviour (TPB) [23]. TPB predicts deliberate behaviour and has been used extensively in the healthcare field [36]. Importantly, TPB describes intention as the combined result of three elements: the individual’s attitudes, subjective norms and perceived behavioural control. The individual’s attitude to behaviour is to some degree valued either positively or negatively and is determined by behavioural beliefs and the subjective likelihood that the behaviour will produce a given result. Subjective norm is the perceived social pressure to perform the behaviour and is determined by normative beliefs, where the prominence of the norm and willingness to comply are of importance. Perceived behavioural control consists of the individual’s perception of his/her ability to perform the behaviour and is determined by control beliefs, which are factors that can facilitate or hinder performance of the behaviour. If these three elements are generally positive, the individual will have the intention to perform the behaviour but if the behaviour is beyond the individual’s control the behaviour does not occur and perceived behavioural control thus has a direct impact on behaviour [23,26].

The theory-based educational intervention focused on Ajzen’s [23] three elements mentioned above in an attempt to increase the intention among RNs to perform the desired behaviour (Figure 1). The theory-based educational intervention included a workshop, the introduction of a pain management pocket guide and a change in the standard ward routines to also include daily systematic pain assessments using VAS for patients experiencing cancer pain. The curriculum for the workshops was developed from TPB [23] with the aim of supporting behavioural change. The curriculum included three distinct elements: beliefs about the factors that help or hinder behaviour, beliefs about the normative expectations and beliefs about the likely impact of the behaviour, which Ajzen [23] suggests control human behaviour. Consequently, the educational activities in the workshop focused on these components to achieve greater intention (Figure 1). The workshop, lasting 120 minutes, was conducted in two sessions. Twenty-five RNs were divided into two groups, one with 11 RNs and one with 14, where each group attended one of the sessions. The research team considered this to be an appropriate group size for educational activities. The workshop started with a brief introduction and the RNs were then divided into three smaller groups and given a unique patient scenario involving surgical cancer where incorrect and correct practices in pain management, i.e. evidence-based practices, were introduced. Each group worked through the scenarios using their own personal experience and knowledge. The discussion was then reflected on together with both groups with a focus on the knowledge and attitudes that emerged. Two university lecturers involved in the study helped the RNs to reflect on and summarise discussions. The content of the workshops and the pocket-size guide handed out to the RNs were based on the Scottish Intercollegiate Guidelines Network [37] guidelines for control of pain in adult patients with cancer as well as on knowledge derived from the literature searches for this study.

In order to support parts of the theory-based educational intervention, more specific “perceived behavioural control” (Figure 1) a practical component was also included in the form of daily systematic pain assessment. This was to be performed by the RNs for all patients on the intervention ward with a cancer diagnosis. In order to assess the patients’ current pain intensity, a Visual Analogue Scale (VAS) [38] was introduced and handed out to the RNs. VAS comprises a 10 cm line measuring pain intensity with two extreme limits. Pain intensity is marked between the two extremes, ranging from no pain to worst imaginable pain [38]. VAS can be used to assess acute pain as well as evaluate current pain treatment [39]. Test-retest reliability in four studies involving adults with cancer pain showed an average coefficient of r = 0.80 [40]. The RNs’ daily pain assessment was performed in conjunction with the ward’s routine assessment of vital parameters. VAS was to be assessed three times a day between three shifts: 4 am–6 am, 1 pm–3 pm and 7 pm–9 pm.

### Statistical analysis

Descriptive and comparative statistics [41] were used to analyse demographic data, percentages and the number of correct answers from the NKAS. Descriptive statistics was used for single items from NKAS. According to the authors of the NKAS [30], the instrument is found to be most useful for analysing the data in terms of the percentage of complete scores as well as analyses of individual items. An analysis was therefore performed using all valid data from individual items in the NKAS in accordance with the intention-to-treat principle [42]. When analysing a complete the NKAS score, only those RNs who completed the NKAS in full were included. A chi-squared test [43] was used to analyse categorical data, i.e. differences between RNs in terms of educational level and amount of pain education. The Mann–Whitney U-test [43] was used to compare mean scores between the intervention ward and the control ward, i.e. work experience as an RN and age. The Mann–Whitney U-Test was also used to test the differences in mean NKAS scores between the intervention ward and the control ward at the three measurement points. The Wilcoxon sign test [43] was used to analyse the primary outcome and sub-items 35 and 36 in the NKAS in paired groups between measurement points T1 and T2. The statistical significance was set at p < 0.05. The internal consistency for the NKAS was calculated using Cronbach’s alpha coefficient [32]. Data were analysed using SPSS, version 20. Sample size calculations for the primary outcome, the NKAS, were based on the estimation of a five-point change in scores [44] for the RNs on the intervention ward. A significance level ᾳ of 0.05 (two-sided) and a power of 80% required 12 RNs per group.

### Ethical considerations

This study was conducted in compliance with the established ethical guidelines of the Declaration of Helsinki [45]. According to the Swedish Ethical Review Involving Humans Act [46], this study did not need ethical clearance by a Regional Ethical Review Board, although ethical guidance and advisory opinions were sought and received from the Ethical Advisory Board in South-East Sweden (Ref. 61–2011). All participants received verbal and written information about the study and were informed of their right to withdraw at any time. Data were stored securely and anonymously in compliance with the Data Protection Act [47].

## Results

A total of 40 RNs from both wards completed the NKAS, resulting in an overall response rate of 60% at T1 (Table 1). Initially, 60 RNs from both wards were eligible to participate, 33 RNs on the intervention ward and 27 RNs on the control ward. There were no significant differences between the participating RNs on the intervention ward or control ward with regard to age, gender or working experience. However, as regards education, significantly more RNs on the intervention ward than on the control ward had a nursing degree (Table 1).

**Table 1:** Characteristics of the two groups of participating RNs (n = 40) at T1

	**Intervention ward**	**Control ward**	***p*-value**
*RNs (n)*	25	15	
*Gender* Female (%)	100	100	
*Age* (mean/SD)	38.0 (±12.0)	36.8 (±9.6)	0.751^2^
<30 years (%)	32	20	
30-39 years	36	40	
≥40 years	32	40	
*Working experience* (mean/SD)	10.2 (±12.4)	9.4 (±7.5)	0.815^2^
<5 years (%)	44	27	
5-10 years	28	40	
>10 years	28	33	
*Educational level* (%)			0.008^3^
Diploma	24	33	
Degree	76	67	
*Pain education* (%)			0.493^3^
Pain course, 15 ETCS	12	20	
*Primary outcome, NKAS*^*1*^ (Correct answer scores/SD)	25.5 (±4.3)	26.2 (±2.9)	0.795^2^

^*1*^ The internal dropout for NKAS at T1 was three respondents from the intervention ward and five respondents from the control ward.

^2^ The Mann–Whitney U-Test was used to compare mean scores with regard to age, working experience and the NKAS between the intervention ward and the control ward.

^3^ The Chi-squared test was used to analyse categorical data, i.e. education level and pain education.

The reasons for RNs (n = 8) from the intervention ward dropping out at T1 were: sick leave (n = 1), working night shift (n = 2), not being able to leave the ward at the time of the workshop due to low staffing (n = 2) and lack of time to fill in the NKAS (n = 3). Consequently, 76% (n = 25) of the eligible RNs (n = 33) on the intervention ward participated in the workshop and completed the NKAS at T1 (Table 1). However, of these 25 RNs, three failed to answer all the NKAS items, resulting in their exclusion from any statistical analyses based on a completed NKAS (n = 22) but not from analyses of the individual NKAS items (Table 2).

**Table 2:** Overview attrition rate between T1 and T3

	**Intervention ward**	**Control ward**	**Total sample**
**T1**			
Number in analysis - complete NKAS	22	10	32
Number in analysis - individual NKAS items	25	15	40
**T2**			
Number in analysis - complete NKAS	6	7	13
Number in analysis - individual NKAS items	6	9	17
**T3**			
Number in analysis - complete NKAS	4	6	10
Number in analysis - individual NKAS items	4	9	13

On the control ward, 56% (n = 15) of the eligible RNs (n = 27) completed the NKAS at T1. The reasons for RNs from the control ward dropping out at T1 were: sick leave (n = 2), lack of time (n = 6) and forgetting to fill in the NKAS (n = 4). Five RNs failed to answer all the NKAS items, leading to the same procedure as described above with only five RNs being included in statistical analyses of individual items and just 10 RNs in analyses based on a completed the NKAS (Table 2).

At baseline, T1, the number of correct answers in the total sample of RNs completing the NKAS (n = 32) resulted in a mean score of 25.7 points (SD ±3.85) and a 67.6% correct answer rate. The percentage of correct answers for the NKAS for RNs (n = 22) on the intervention ward ranged between 42.0% and 84.0% and for the RNs (n = 10) on the control ward between 56.8% and 81.6%. There were no significant differences between the RNs on the intervention and control wards with regard to the number of correct answers in the NKAS (Table 1).

At T2, the number of correct answers in the total sample of RNs (n = 13, see Table 2 for attrition) completing the NKAS resulted in a score of 27.6 points (SD ±3.5) and a 72.6% correct answer rate. The percentage of correct answers in the NKAS for RNs (n = 6) on the intervention ward ranged between 63.2% and 89.5% and for the RNs (n = 7) on the control ward between 60.5% and 86.8% (Table 3). There was a significant difference (*p* = 0.028) in the number of correct answers in the NKAS for the intervention ward RNs between T1 to T2 and the number of correct answers, i.e. higher scores, increased by 13.6% (range 2.6-31.7%). No significant differences were found between T1 and T2 for the RNs on the control ward although the number of correct answers increased by 1.5% and the change in the NKAS score ranged from −5.3% to 12.2% (Table 3).

**Table 3:** Primary outcome, NKAS, mean percentages at baseline (T1) and four weeks after the start of the theory-based intervention (T2)

**Primary outcome NKAS**^**1**^	**T1**			**T2**			**p-value**
	**Mean**	**SD**	**95% CI**	**Mean**	**SD**	**95% CI**	
Intervention ward	67.0	±11.2	62.0-71.9	73.7	±9.6	63.7-83.7	0.028^1,2^
Control ward	67.8	±8.1	62.4-73.3	71.8	±9.5	63.1-80.1	0.671^1,2^

^**1**^ The internal dropout for NKAS between T1 and T2 was 16 respondents from the intervention ward and three respondents from the control ward.

^2^ The Wilcoxon sign test was used to analyse primary outcome NKAS in paired groups between T1 and T2.

For two items, 26 and 36 (Table 4), the correct answer rate among the total sample of RNs (n = 40) was below 25% at T1. Item 26 concerned the likelihood of the patient developing clinically significant respiratory depression. Only 13% of the RNs (Four RNs on the intervention ward and one RN on the control ward) answered the correct incidence rate and the other RNs (n = 35) overstated the incidence rate. At T2, two of the six remaining RNs from the intervention ward answered the correct incidence rate for clinically significant respiratory depression and the remainder (n = 4) overstated the incidence rate.

**Table 4:** Overview of percentage of correct NKAS answers by RNs (n = 40) at baseline (T1)

**True or false statements**	**Intervention ward**		**Control ward**		**Both wards**
	**(%)**	**(n)**	**(%)**	**(n)**	**(%)**
**1.** Vital signs are always reliable indicators of the intensity of a patient’s pain.	72	18	60	9	68
**2.** Because their nervous system is underdeveloped, children under two years of age have decreased pain sensitivity and limited memory of painful experiences.	68	17	80	12	73
**3.** Patients who can be distracted from pain usually do not have severe pain.	68	17	60	9	65
**4.** Patients may sleep in spite of severe pain.	44	11	47	7	45
**5.** Aspirin and other non-steroidal anti-inflammatory agents are NOT effective analgesics for painful bone metastases.	36	9	53	8	43
**6.** Respiratory depression rarely occurs in patients who have been receiving stable doses of opioids over a period of months.	68	17	67	10	68
**7.** Combining analgesics that work using different mechanisms (e.g. combining an opioid with an NSAID) may result in better pain control with fewer side effects than using a single analgesic agent.	96	24	100	15	98
**8.** The usual duration of analgesia of 1–2 mg morphine IV is 4–5 hours.	60	15	67	10	63
**9.** Opioids should not be used in patients with a history of substance abuse.	64	16	47	7	58
**10.** Morphine has a dose ceiling (i.e. a dose above which no greater pain relief can be obtained).	84	21	67	10	78
**11.** Elderly patients cannot tolerate opioids for pain relief.	96	24	93	14	95
**12.** Patients should be encouraged to endure as much pain as possible before using an opioid.	100	25	100	15	100
**13.** Children less than 11 years old cannot reliably report pain so nurses should rely solely on the parents’ assessment of the child’s pain intensity.	96	24	87	13	93
**14.** Patients’ spiritual beliefs may lead them to think pain and suffering are necessary.	68	17	93	14	78
**15.** After an initial dose of opioid analgesic is given, subsequent doses should be adjusted in accordance with the individual patient’s response.	84	21	100	15	90
**16.** Giving patients sterile water by injection (placebo) is a useful test to determine if the pain is real.	88	22	93	14	90
**17.** If the source of the patient’s pain is unknown, opioids should not be used during the pain evaluation period, as this could mask the ability to correctly diagnose the cause of pain.	60	15	27	4	48
**18.** Anticonvulsant drugs such as gabapentin (Neurontin) provide optimal pain relief after a single dose.	100	25	93	14	98
**19.** Benzodiazepines are not effective pain relievers unless the pain is due to muscle spasm.	40	10	40	6	40
**20.** Narcotic/opioid addiction is defined as a chronic neurobiological disease, characterised by behaviours that include one or more of the following: impaired control over drug use, compulsive use, continued use despite harm, and craving.	76	19	87	13	80
Multiple choice questions*					
**21.** The recommended route of administration of opioid analgesics for patients with persistent cancer-related pain is:	44	11	33	5	40
**22.** The recommended route administration of opioid analgesics for patients with brief, severe pain of sudden onset such as trauma or postoperative pain is:	84	21	100	15	90
**23.** Which of the following analgesic medications is considered the drug of choice for the treatment of prolonged moderate to severe pain for cancer patients?	96	24	93	14	95
**24.** Which of the following IV doses of morphine administered over a 4-hour period would be equivalent to 30 mg of oral morphine given q 4 hours?	28	7	40	6	33
**25.** Analgesics for post-operative pain should be given initially:	84	21	67	10	80
**26.** A patient with persistent cancer pain has been receiving daily opioid analgesics for two months. Yesterday the patient was receiving morphine 200 mg/hour intravenously. Today he has been receiving 250 mg/hour intravenously. The likelihood of the patient developing clinically significant respiratory depression in the absence of new comorbidity is:	16	4	7	1	13
**27.** The most likely reason a patient with pain would request increased doses of pain medication is:	76	19	100	15	85
**28.** Which of the following is useful for treatment of cancer pain?	44	11	40	6	43
**29.** The most accurate judge of the intensity of the patient’s pain is:	96	24	87	13	93
**30.** Which of the following describes the best approach for cultural considerations in caring for patients in pain:	88	22	80	12	85
**31.** How likely is it that patients who develop pain already have an alcohol and/or drug abuse problem?	44	11	20	3	36
**32.** The time to peak effect for morphine given IV is:	84	21	80	12	83
**Items**	**Intervention ward**		**Control ward**		**Both wards**
	**(%)**	**(n)**	**(%)**	**(n)**	**(%)**
**33.** The time to peak effect for morphine given orally is:	64	16	67	10	65
**34.** Following abrupt discontinuation of an opioid, physical dependence is manifested by the following:	48	12	47	7	48
**Case studies**					
**35.** Patient A: Andrew is 25 years old and this is his first day following abdominal surgery. As you enter his room, he smiles at you and continues talking and joking with his visitor. Your assessment reveals the following information: BP = 120/80; HR = 80; R = 18; on a scale of 0 to 10 (0 = no pain/discomfort, 10 = worst pain/discomfort) he rates his pain as 8. On the patient’s record you must mark his pain on the scale below. Circle the number that represents your assessment of Andrew’s pain.	48	12	40	6	46
**36.** Your assessment, above, is made two hours after he received morphine 2 mg IV. Half-hourly pain ratings following the injection ranged from 6 to 8 and he had no clinically significant respiratory depression, sedation, or other untoward side effects. He has identified 2/10 as an acceptable level of pain relief. His physician’s order for analgesia is “morphine IV 1–3 mg q1h PRN pain relief”. Check the action you will take at this time.	20	5	27	4	23
**37.** Patient B: Robert is 25 years old and this is his first day following abdominal surgery. As you enter his room, he is lying quietly in bed and grimaces as he turns in bed. Your assessment reveals the following information: BP = 120/80; HR = 80; R = 18; on a scale of 0 to 10 (0 = no pain/discomfort, 10 = worst pain/discomfort) he rates his pain as 8. On the patient’s record you must mark his pain on the scale below. Circle the number that represents your assessment of Robert’s pain:	60	15	53	8	59
**38.** Your assessment, above, is made two hours after he received morphine 2 mg IV. Half-hourly pain ratings following the injection ranged from 6 to 8 and he had no clinically significant respiratory depression, sedation, or other untoward side effects. He has identified 2/10 as an acceptable level of pain relief. His physician’s order for analgesia is “morphine IV 1–3 mg q1h PRN pain relief”. Check the action you will take at this time:	40	10	40	6	40

*A full description of alternative answers to multiple-choice questions is presented in the NKAS (Ferrell & McCaffery [30]).

Item 36 was a patient case scenario question regarding administration of morphine and was an extension of item 35. Both items 35 and 36 reflected how the RNs viewed the patient’s own perceived pain. For item 35, 46% of the RNs (n = 40) answered correctly, which meant that 18 RNs rated the case study’s patient’s pain as 8, which was in accordance with the patient’s own perceived pain intensity. Even if not statistically significant (*p* = 0.068), the RNs (n = 6) on the intervention ward were found at T2 to rate the case study patient’s pain closer to the patient’s own perceived pain with an increased mean value of 1.83 compared to their rating of the patient’s perceived pain at T1. RNs on the control ward (n = 9) also had an increase in their rating of the case study patient’s pain between T1 and T2 by a mean of 0.94 (*p* = 0.068).

For item 36, only 23% of the RNs (n = 9) on both wards at T1 (Table 4) stated the correct amount of a ‘when needed’ dosage of morphine as 3mg, as recommended in the NKAS answer book. The RNs on the intervention ward (n = 6) were found to increase the ‘when needed’ dosage of morphine in their answers from T1 to T2 towards the accurate amount of morphine, with an increased mean of 0.83 mg more morphine even though it was not statistically significant (*p* = 0.102). The control ward RNs (n = 9) were also found to increase the dosage of morphine in their answers from T1 to T2 by a mean of 0.22 mg more morphine, but not statistically significant (*p* = 0.317).

The RNs on the intervention ward who completed T2 and T3 (Table 2) retained their rating of the patient’s pain (Item 35) at T3, and this was in accordance with the case study patient’s own perceived pain rating. From T2 to T3 the RNs on the intervention ward (Table 2) were found to both refrain from stating the correct dosage of morphine (Item 36) but also to reduce, as one of the RNs reduced her answer about dosage by 1mg from the correct amount of 3mg.

At T3, the number of correct answers in the total sample of RNs (n = 10), see Table 2 for attrition) completing the NKAS resulted in a score of 26.5 points (SD ±3.5) and a 69.5% correct answer rate. The percentage of correct answers in the NKAS for RNs (n = 4) on the intervention ward ranged between 71.1% and 76.3% and for the RNs (n = 6) on the control ward between 55.3% and 81.6%. There was no significant difference in the number of correct NKAS answers, i.e. scores for the RNs on the control ward between T1 and T2 (*p* = 0.671), T2 and T3 (*p* = 0.144) or T1 and T3 (*p* = 0.108). No statistical analysis could be performed for the RNs on the intervention ward to compare their NKAS scores between T2 and T3 due to the high attrition rate throughout the study and particularly at T3 (Table 2).

Of the RNs (n = 10) who completed the NKAS in full at T3, all were female, seven (70%) had a degree and three (30%) had a diploma. Mean working experience was 11.1 years (SD ±11.9) and the mean age was 41.9 years (SD ±13.3). One RN (10%) had completed a pain course comprising 15 ECTS and the remainder (n = 9) had not taken any pain course. This can be compared with the total sample of RNs (n = 40) at T1, where all RNs were female, 29 (72.5%) had a degree and eleven (27.5%) had a diploma. Mean working experience was 9.9 years (SD ±10.7) and the mean age was 37.5 years (SD ±11.0). Six RNs (15%) had completed a pain course comprising 15 ECTS and the remainder (n = 34) had not taken any pain course.

## Discussion

This study aimed to investigate whether an intervention targeting RNs (Figure 1) and consisting of three components – a theory-based educational workshop, the introduction of a pain management pocket guide and daily systematic pain assessments – could be effective in changing RNs’ knowledge and attitude regarding cancer pain and pain management. The findings indicated that it is possible to achieve a significantly positive change in both knowledge and attitude with the help of a brief interactive workshop (Table 3). Unfortunately, the findings are only based on a positive change measured after four weeks and do not reveal if there would be a lasting positive change in attitude or knowledge among the participating RNs. Other studies investigating different types of educational interventions [44,48] have been shown to bring about a positive change in the RNs’ knowledge and attitudes regarding pain although these educational interventions were not based on any theory of behavioural change and were extensive in terms of staffing requirements. This might not be the right solution or even feasible for RNs on wards caring for patients with cancer diagnoses since time and resources can be scarce for educational initiatives in healthcare [49].

Even though the Swedish version of the NKAS had two items less than the English version developed by Ferrell and McCaffery [30], the RNs at T1 had a higher correct answer rate than RNs in other studies [15,16]. The RNs (n = 32) were found to have a 67.6% (SD ±10.2) correct answer rate in the NKAS. When compared with two European studies, including RNs in the same context, their results showed an overall mean score of 45.1% (SD ±19.3) [15] and 55.0% (SD ±25.9) [16]. Although there was a higher mean score, these results still show deficiencies in knowledge and attitudes amongst RNs and these findings are consistent with other studies involving RNs working in different clinical contexts [50-52]. In this study’s current sample, all RNs are female. However, this overrepresentation reflects the present-day gender distribution within the Swedish nursing profession (2010) – 91% of Swedish RNs are female and 9% male [53].

The RNs at T1 on the intervention ward were found in their answers to frequently underrate the fictional patient’s perceived pain intensity. This was apparent in the two case scenario questions related to believing the patient’s own experienced pain (Items 35 and 37, Table 4), which showed that only 48% of the RNs believed the patient when he/she did not show any non-verbal signs of pain and 60% when they showed non-verbal signs. It is a problem when half of the RNs on the intervention ward did not believe the patient’s perceived pain intensity. Furthermore, patients experiencing cancer pain might not always show non-verbal signs, i.e. bodily signs. Because pain is a complex interaction between pathophysiological and biopsychosocial factors, the only ones who can rightfully rate the pain experience are the patients themselves [54]. However, at T2, following the educational intervention, the RNs (n = 6) on the intervention ward were found to rate the case study patient’s pain (Item 35, Table 4) closer to the patient’s own rating by a mean value of 1.83, although not significantly.

A substantial number of the RNs on the intervention ward were found to answer items 36 and 38, which deal with morphine dosage, with an insufficient amount of morphine, which would in turn result in undertreatment of the patient’s pain. This was seen particularly in item 36 (Table 4), where at T1 only 20% of the RNs on the intervention ward would have administered a sufficient amount of morphine to the case study patient. However, from T1 to T2 the RNs (n = 6) on the intervention ward, after undergoing the educational intervention, were found to increase the dosage of morphine in their answers towards the correct amount of morphine, although not significantly. Another important barrier to adequate pain management is the exaggeration of certain opioid side effects [55]. One of these side effects is respiratory depression, which is infrequent amongst patients taking opioids regularly and over a long period [56]. Despite this, only 16% of the RNs on the intervention ward stated the correct incidence rate (Item 26, Table 4) and the remainder overstated the incidence rate of respiratory depression. According to Hutton and colleagues [55], the fear of respiratory depression can lead to an inadequate dose of morphine being administered by RNs.

Findings from this study have important implications for nursing and cancer pain management. RNs often function as advocates for their patients and must be knowledgeable, not allowing their own attitudes to influence the patients’ pain management negatively. There is an acute need for educational interventions in this area since these findings indicate that there may be deficiencies that could affect the patients’ well-being. This kind of educational activity could be interactive and be performed on a regular basis in an attempt to maintain the positive effects. Before designing the educational initiatives that are needed, it is essential to assess the clinic’s own problem areas to know where to strengthen the efforts that are being made.

### Methodological considerations

This study used a quasi-experimental design and therefore did not check for selection bias [29]. However, randomisation and blinding of RNs are not always possible when conducting a quality improvement study in healthcare with a limited budget. According to Thompson and Panacek [57], the researcher is obligated to appraise the scientific rigour in relation to the context and available resources. Another limitation is the lack of measurement of how well the RNs adhered to the principles of the intervention. No checks were made to see if the RNs on the intervention ward followed the recommendations regarding cancer pain management or if they performed the daily systematic pain assessments. This has affected the generalisability of the findings. An additional limitation was the attrition rate [29] with a high dropout followed by a low NKAS response rate from T1 to T3 (Table 2). This limitation might be explained by the organisational difficulties that occurred during the study period, i.e. short staffing, changes in the organisational structures and high bed occupancy. Despite the assurance from all RNs on the intervention ward that they would complete all their questionnaires, this was not done. Even though data were treated in accordance with the intention-to-treat principle [42], this only helped when individual items were analysed. This limitation has affected the external validity [29] and the generalisability of the findings, since data analysis of primary outcome at T3 could not be completed because of the high attrition rate (Table 2). Allowing RNs time to fill in their questionnaires away from their patient care responsibilities might have reduced the dropout rate. Then again, these kinds of limitations are not uncommon when conducting experimental research in healthcare and according to Rycroft‒Malone [58] the nursing context involves numerous interactions that are likely to govern the outcome of the study. As exemplified in a study by van der Helm and colleagues [59], well-designed implementation that takes into account leadership support, local barriers, simplicity and co-operating nurses with a perceived need to change, did not lead to indefinitely successful implementation because of the ever-changing healthcare environment. Part of the challenge lies in conducting experimental research within a health organisation with a lack of resources but with a dire need to improve cancer pain management.

This study initially had a secondary aim, which was to investigate whether the intervention would also have a positive impact on the hospital patients’ perception of their cancer pain [60]. Unfortunately, this aim was not fulfilled because of a lack of vital data before the end of this study.

## Conclusion

The primary aim of this study was to investigate whether a theory-based educational intervention could change RNs’ knowledge and attitudes to cancer pain and pain management, both four and 12 weeks after the start of the intervention. This study indicated that a theory-based educational intervention consisting of a brief workshop, including interactive learning activities, distribution of a pain management pocket guide and daily systematic pain assessment, resulted in a positive improvement in RNs’ knowledge and attitudes regarding cancer pain four weeks after the start of the intervention. However, research into this kind of theory-based educational intervention with interactive learning is sparse and this area needs to be explored further and with the assessment of the carry-over effect on the patients taken into account.

## Abbreviations

NKAS: Nurses’ knowledge and attitudes survey regarding pain; RN: Registered nurse; TPB: Theory of planned behaviour; VAS: Visual analogue scale.

## Competing interests

The authors declare that they have no competing interests.

## Authors’ contributions

MG and GB conceived and designed the study and obtained funding. MG was responsible for data collection and data analysis. MG and GB drafted the manuscript and both read and approved the final manuscript.

## Pre-publication history

The pre-publication history for this paper can be accessed here:

http://www.biomedcentral.com/1472-6963/13/328/prepub
